# miR-24-3p Dominates the Proliferation and Differentiation of Chicken Intramuscular Preadipocytes by Blocking *ANXA6* Expression

**DOI:** 10.3390/genes13040635

**Published:** 2022-04-02

**Authors:** Zhongzhen Lin, Yuan Tang, Zhiqiang Li, Jingjing Li, Chunlin Yu, Chaowu Yang, Li Liu, Yan Wang, Yiping Liu

**Affiliations:** 1 Farm Animal Genetic Resources Exploration and Innovation Key Laboratory of Sichuan Province, Sichuan Agricultural University, Chengdu 611130, China; zzlin599@163.com (Z.L.); 2021202030@stu.sicau.edu.cn (Y.T.); 2020202024@stu.sicau.edu.cn (Z.L.); jingjingyi11@126.com (J.L.); liuli55@stu.sicau.edu.cn (L.L.); wangyan519@sicau.edu.cn (Y.W.); 2 Animal Breeding and Genetics Key Laboratory of Sichuan Province, Sichuan Animal Science Academy, Chengdu 610066, China; yuchunlin1984@sina.com (C.Y.); cwyang@foxmail.com (C.Y.)

**Keywords:** miRNAs, *ANXA6*, intramuscular fat, lipogenesis, avian

## Abstract

Intramuscular fat (IMF) is one of the crucial factors determining meat quality. IMF deposition depends on the hyperplasia and hypertrophy of intramuscular preadipocytes, in which genes and noncoding RNAs play an important regulatory role. According to previous transcriptome analysis, *ANXA6* and miR-24-3p were identified as involved in lipid metabolism in breast muscle. In this study, we further investigated their function in the proliferation and differentiation of chicken intramuscular preadipocytes. The results indicated that overexpression of *ANXA6* inhibited proliferation and promoted differentiation of intramuscular preadipocytes, while knockdown of *ANXA6* promoted cell proliferation and inhibited adipogenic differentiation. miR-24-3p was proved to directly bind to the 3′ untranslated region (3′UTR) of *ANXA6* by dual-luciferase reporter assay. The regulatory effect of miR-24-3p on the proliferation and differentiation of intramuscular preadipocytes was opposite to that of *ANXA6*. Besides, the overexpression vector of *ANXA6* eliminated the impact of miR-24-3p mimics on intramuscular preadipocytes. In brief, we revealed that miR-24-3p promoted proliferation but inhibited differentiation of intramuscular preadipocytes by blocking *ANXA6* expression, thus dominating IMF deposition in broilers. These findings may provide a novel target for improving chicken meat quality.

## 1. Introduction

In the past few decades, the breeding of meat-type animals has mainly aimed at improving the growth rate, feed conversion rate, and lean meat rate. This breeding strategy is conducive to promoting the production efficiency of animal husbandry but is often accompanied by a decline in meat quality [1]. With the growth in living standards, consumers are no longer satisfied with the quantity of livestock products, yet have an increasing requirement for the texture and flavor constituted by cooking. Intramuscular fat (IMF) is one of the crucial factors determining meat quality. Its appropriate deposition not only ameliorates muscle tenderness but also contributes to enhancing the flavor and juiciness of meat [2]. The unique marble characteristic of IMF also help consumers make purchase decisions [3].

Intramuscular preadipocytes, derived from the adipogenic differentiation process of mesenchymal stem cells, begin to store lipid substances in muscle tissue after differentiation into mature adipocytes [4]. Therefore, the content of IMF largely depends on the hyperplasia and hypertrophy of intramuscular preadipocytes, which are controlled by many factors, including various genes and noncoding RNAs [5].

microRNAs (miRNAs) are endogenous small noncoding RNAs composed of 22–24 nucleotides [6]. As single-stranded RNAs, miRNAs regulate the translation of messenger RNAs (mRNAs) at post-transcriptional levels by binding to their 3′ untranslated region (3′UTR) [7]. miRNAs are closely involved in the treatment of a variety of diseases and the maintenance of human health [8]. For animal husbandry, miRNAs play a critical role in regulating growth performance, meat quality, and reproductive traits of livestock [9,10]. Thus, it is of great practical value to explore the function and regulatory mechanism of different miRNAs. miR-24-3p was reported to participate in lipid metabolism in chicken liver [11]. The expression of miR-24-3p declined significantly after the differentiation of bovine preadipocytes [12].

Chicken is an important agricultural animal, which provides abundant protein for our diet. The market share of chicken is second only to pork in China [13], implying that elevating the meat quality of chicken may bring tremendous economic benefits. Daheng broiler is a specialized meat-type breed with stable production performance and distinctive meat quality after moderate selection. The live weight and breast muscle weight of 90-day-old male individuals can reach 1816.00 g and 188.54 g, while female individuals reach 1460.00 g and 137.50 g, respectively [14]. Our previous transcriptome analysis presented that annexin A6 (*ANXA6*) was differentially expressed in the breast muscles of Daheng broilers at different ages, and enriched in gene ontology (GO) terms such as phospholipid binding, lipid binding, and cell differentiation [15]. miR-24-3p was predicted to target the 3′UTR region of *ANXA6*. Consequently, in this study, we investigated the effects of *ANXA6* and miR-24-3p on the proliferation and differentiation of chicken intramuscular preadipocytes. These results will provide novel insight for IMF deposition and chicken quality improvement.

## 2. Materials and Methods

### 2.1. Experimental Animals

All animal experiments in this study were approved by the Institutional Animal Care and Use Committee of Sichuan Agricultural University (approval no. DKY2020202025). A total of 200 female Daheng broilers were raised at the Sichuan Daheng Poultry Breeding Co., Ltd. (Chengdu, China) to obtain breast muscle at age of 60 day, 90 day, 120 day, 150 day, 180 day, 240 day, and 300 day. Eleven tissue samples, including heart, liver, kidney, breast muscle, leg muscle, subcutaneous fat, abdominal fat, brain, gizzard, glandular stomach, and small intestine, were collected from three 90-day-old female Daheng broilers. These samples were stored immediately in liquid nitrogen for further analysis. Besides, sufficient 14-day-old chicks were prepared for primary intramuscular preadipocytes isolation. All experimental birds were euthanized by cervical dislocation.

### 2.2. Cell Isolation, Culture, and Differentiation

Primary intramuscular preadipocytes were isolated from breast muscle tissues of 14-day-old chicks. Briefly, after removing the fascia and connective tissues, breast muscles were cut to pieces by ophthalmic scissors. Collagenase type I and type II (Biofroxx, Heidelberg, Germany) were used to digest tissue homogenate for 1.5 h, which were then passed through cell strainers (Biologix, Jinan, China) with pore sizes of 70 μm and 45 μm. The cell precipitate was gained via centrifugation at 1000 r/min for 10 min. Subsequently, these cells were maintained in DMEM/F12 medium (Gibco, Grand Island, NY, USA) supplemented with 10% fetal bovine serum (Hyclone, Logan, UT, USA) and 1% penicillin/streptomycin (Invitrogen, Carlsbad, CA, USA) in an incubator with 37 °C, 5% CO_2_, and a humidified atmosphere. Because the adherent time of muscle satellite cells exceeded 3 h, after differential adherence for 2 h, the supernatant was removed and replaced with fresh complete medium to obtain relatively pure preadipocytes. Once the preadipocytes grew confluent, a differentiation medium containing 10 μg/mL insulin, 250 μM oleic acid, 0.5 mM 3-isobutyl-1-methylxanthine, and 1 μM dexamethasone was replaced to induce differentiation for 2 day. Then, a maintenance medium containing 10 μg/mL insulin and 250 μM oleic acid was replaced to maintain differentiation for 2 day. Finally, the complete medium was replaced every 2 day until the cells were differentiated into mature adipocytes. The differentiation time lasts for 10 day. All trials performed on cells contained at least three biological replicates.

### 2.3. Cell Transfection

Briefly, the RNA oligonucleotides or plasmids were diluted in Lipofectamine 3000 (Invitrogen, Carlsbad, CA, USA) and Opti-MEM medium (Gibco, Grand Island, NY, USA) according to the manufacturer’s instructions. Then, these mixed liquids were added to cell culture plates of different specifications in corresponding proportions. After incubation for 6–8 h, fresh complete medium was replaced in the cell culture plates. RNA oligonucleotides for miR-24-3p, including mimics of the negative control (NC), miR-24-3p mimics, inhibitor NC, and miR-24-3p inhibitor, as well as three small interfering RNAs (siRNAs) for *ANXA6*, were designed and synthesized by GenePharma (Shanghai, China) (Supplementary Table S1). Overexpression vector pcDNA3.1+*ANXA6* was constructed by two restriction enzymes, BamHI and XhoI.

### 2.4. RNA Extraction, cDNA Synthesis, and Quantitative Real-Time PCR

Total RNA was extracted from cells and tissues using Trizol reagent (TaKaRa, Dalian, China), and its concentration and purity were determined by NanoDrop 2000C spectrophotometer (Thermo, San Jose, CA, USA). Subsequently, cDNA was synthesized through reverse transcription of mRNA by PrimeScript RT Reagent Kit (TaKaRa, Dalian, China). CFX Connect Real-Time System was used to conduct quantitative real-time PCR (qPCR). Relative expression was calculated by the 2^−ΔΔCt^ method, with *GAPDH* and *U6* being reference gene. All the primers used in qPCR assay were devised from Prime Premier 6 (Premier Biosoft, Palo Alto, CA, USA) and presented in Supplementary Table S2.

### 2.5. Western Blotting

Total protein was extracted from cells transfected for 48 h using the Total Protein Extraction Kit (BestBio, Shanghai, China). The concentration of protein samples was detected and homogenized by BCA Protein Quantitative Kit (BestBio, Shanghai, China). Then, briefly, about 25 μg of the protein sample was separated by sodium dodecyl sulfate-polyacrylamide gel electrophoresis. The region containing the target protein was isolated and transferred to polyvinylidene fluoride membranes. After blocking with blocking buffer (Beyotime, Haimen, China) on a decolorization shaker for 1 h at room temperature, the membranes were incubated with primary antibodies overnight at 4 °C, including anti-β-Tubulin (ZenBio, Chengdu, China; 1:5000 dilution), anti-CDK2 (ZenBio, Chengdu, China; 1:1000 dilution), anti-PCNA (ZenBio, Chengdu, China; 1:1000 dilution), anti-PPARγ (ABclonal, Wuhan, China; 1:1000 dilution), and anti-FASN (ZenBio, Chengdu, China; 1:1000 dilution). Subsequently, western wash buffer (Beyotime, Haimen, China) was used to wash the membranes for three times. Secondary antibodies, horseradish peroxidase-conjugated IgG of anti-mouse (ZenBio, Chengdu, China; 1:5000 dilution) or anti-rabbit (ABclonal, Wuhan, China; 1:2000 dilution), were used to incubate the membranes for 1 h at 4 °C. Finally, the Ultra Hypersensitive ECL Chemiluminescence Kit (Beyotime, Haimen, China) was used to visualize the specific protein bands, whose quantitative analysis was performed by Image J 1.8 software.

### 2.6. CCK-8 Assay

Intramuscular preadipocytes were cultured in 96-well plates. After transfection, cell proliferation was monitored using the Cell Counting Kit-8 (Meilun, Dalian, China). Specifically, 10 µL of CCK-8 reagent was added to incubate the cells for 2 h. Optical density (OD) value in each well was detected by Microplate Reader (Thermo, San Jose, CA, USA) at 450 nm after transfection of 12 h, 24 h, 36 h, and 48 h.

### 2.7. EdU Assay

EdU assay was performed on preadipocytes plated in 96-well plates by Cell-Light EdU Apollo567 In Vitro Kit (RiboBio, Guangzhou, China) according to the manufacturer’s protocol after transfection for 12 h. Briefly, 50 μM EdU reagent was added to each well and incubated for 2 h at 37 °C. Cell nuclei were stained by Hoechst 33342 reagent. Biological microscope (Olympus, Tokyo, Japan) was used to capture randomly selected fields, with the number of EdU positive cells and total cells being counted using Image-Pro Plus 6.0 software (Media Cybernetics, Rockville, MD, USA).

### 2.8. Oil Red O Staining

After transfection for 48 h, preadipocytes in 6-well plates were induced to mature adipocytes for 10 d. Subsequently, the adipocytes were washed with PBS once and fixed with 4% paraformaldehyde for 30 min. Fixed cells were washed with distilled water twice, soaked with 60% isopropanol for 5 min, and dyed with Oil Red O (Solarbio, Beijing, China) for 20 min. Then, they were washed with distilled water five times. IX53 biological microscope (Olympus, Tokyo, Japan) was used to capture images of stained cells. In order to quantify lipid droplets, 100% isopropanol was used to dissolve Oil Red O in each well. Finally, OD value was detected at 510 nm.

### 2.9. Target Prediction and Dual-Luciferase Reporter Assay

The upstream miRNAs of *ANXA6* were predicted with miRDB 6.0 software. A complementary pattern diagram of the miR-24-3p and *ANXA6* 3′UTR region was created by RNAhybrid 2.1 software. Dual-luciferase reporter assay was performed in chicken embryo fibroblast line (DF-1 cells). Briefly, the DF-1 cells cultured in 48-well plates were co-transfected with reporter vector (*ANXA6* 3′UTR wild type or mutant type) and mimics NC or miR-24-3p mimics. Following transfection for 48 h, luciferase activity was detected using the Dual Luciferase Reporter Gene Assay Kit (Beyotime, Haimen, China) according to the manufacturer’s instructions.

### 2.10. Statistical Analysis

All data are displayed as mean ± standard error (SEM). One-way ANOVA analysis or unpaired Student’s *t*-test was conducted by SPSS 26.0 software, with the Tukey–Kramer method being used for multiple comparisons. The significant levels were regarded as * *p* < 0.05, ** *p* < 0.01, and ^a–d^ *p* < 0.05.

## 3. Results

### 3.1. Expression Pattern of ANXA6 in Broilers

**Figure 1:**
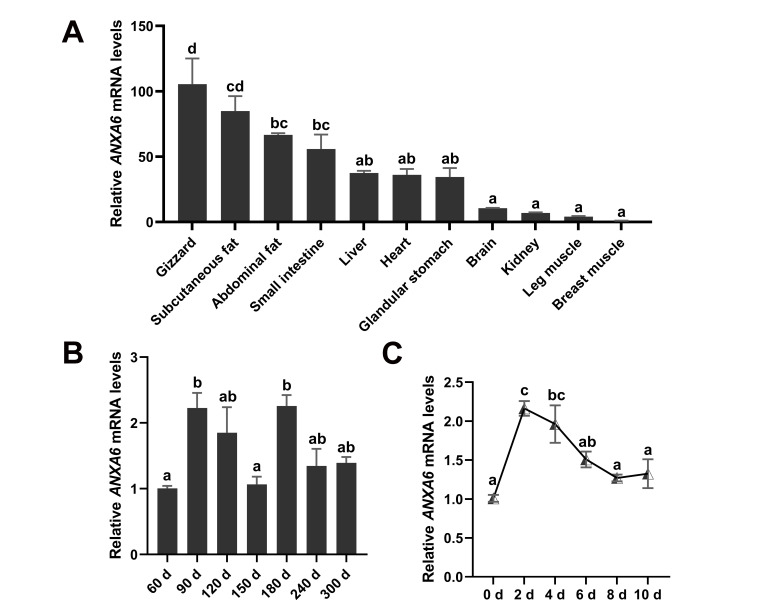
Expression pattern of *ANXA6* in broilers. (**A**) The mRNA levels of *ANXA6* in various broiler tissues. (**B**) Relative expression of *ANXA6* in breast muscle of broilers at different ages. (**C**) The mRNA levels of *ANXA6* during the differentiation process of chicken primary intramuscular preadipocytes into mature adipocytes. All results are presented as mean ± SEM. *n* = 3. ^a–d^ *p* < 0.05.

The tissue expression profile of *ANXA6* in broilers was investigated by qPCR assay, which presented that *ANXA6* was enriched in gizzard, and also highly expressed in subcutaneous fat, abdominal fat, as well as liver (Figure 1A). We determined the mRNA levels of *ANXA6* in chicken breast muscle from 60 d to 300 d after hatching. The results suggested that *ANXA6* reached the highest expression in breast muscle of 90 d and 180 d broilers (Figure 1B). Subsequently, we detected the expression of *ANXA6* during the differentiation process of chicken intramuscular preadipocytes into mature adipocytes, showing that *ANXA6* expression hit peak levels on the second day after differentiation, and then decreased gradually (Figure 1C).

### 3.2. ANXA6 Inhibits the Proliferation of Chicken Intramuscular Preadipocytes

**Figure 2:**
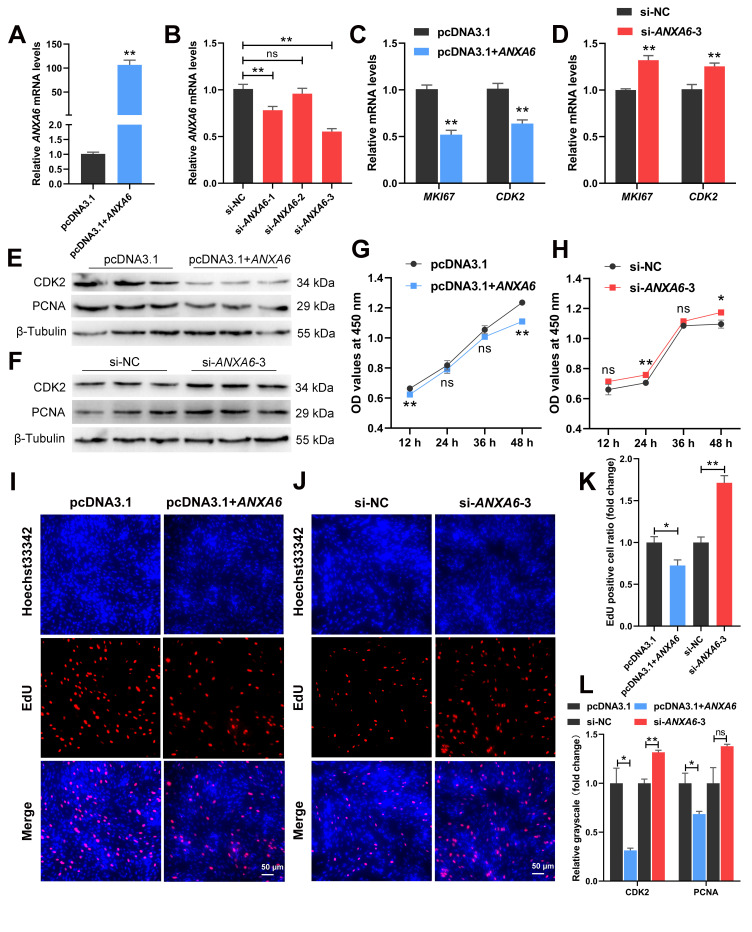
*ANXA6* inhibits the proliferation of chicken intramuscular preadipocytes. (**A**,**B**) Relative expression of *ANXA6* in preadipocytes transfected with overexpression plasmids or siRNAs. (**C**,**D**) Relative mRNA levels of cell-proliferation-related genes (*MKI67* and *CDK2*) after overexpression or interference of *ANXA6*. (**E**,**F**) Relative expression of cell-cycle-associated protein (CDK2 and PCNA) after overexpression or interference of *ANXA6*. (**G**,**H**) Cell growth curves determined by CCK-8 assay at 12 h, 24 h, 36 h, and 48 h following overexpression or interference of *ANXA6*. (**I**,**J**) Proliferation state of preadipocytes assessed by EdU assay after overexpression or interference of *ANXA6*. (**K**) Relative EdU positive cell ratio following overexpression or interference of *ANXA6*. (**L**) Relative grayscale of CDK2 and PCNA proteins relative to β-Tubulin. All results are presented as mean ± SEM. *n* = 3. * *p* < 0.05; ** *p* < 0.01; ^ns^ *p* > 0.05.

To explore the effects of *ANXA6* on chicken intramuscular preadipocytes, the cells were transfected with overexpression vector (pcDNA3.1+*ANXA6*) or siRNAs (siRNA-*ANXA6*-1, siRNA-*ANXA6*-2, and siRNA-*ANXA6*-3). The expression of *ANXA6* was raised in the overexpression group more than 100 times compared with the control group (Figure 2A). Contrarily, both siRNA-*ANXA6*-1 and siRNA-*ANXA6*-3 significantly attenuated the mRNA levels of *ANXA6*, with siRNA-*ANXA6*-3 possessing the highest interference efficiency of 44.7% (Figure 2B). The mRNA levels of cell-proliferation-related genes (*MKI67* and *CDK2*) were reduced or increased after overexpression or interference of *ANXA6* (Figure 2C,D), so did the protein levels of CDK2 and PCNA (Figure 2E,F,L). Nevertheless, the change of PCNA protein expression was not significant after *ANXA6* interference. CCK-8 assay suggested that cell proliferation was inhibited following *ANXA6* overexpression (Figure 2G) but promoted following *ANXA6* knockdown (Figure 2H). EdU assay indicated that EdU positive cell ratio was decreased or increased after *ANXA6* overexpression or interference (Figure 2I–K). Together, these results revealed that *ANXA6* inhibits the proliferation of chicken intramuscular preadipocytes.

### 3.3. ANXA6 Promotes the Differentiation of Chicken Intramuscular Preadipocytes

**Figure 3:**
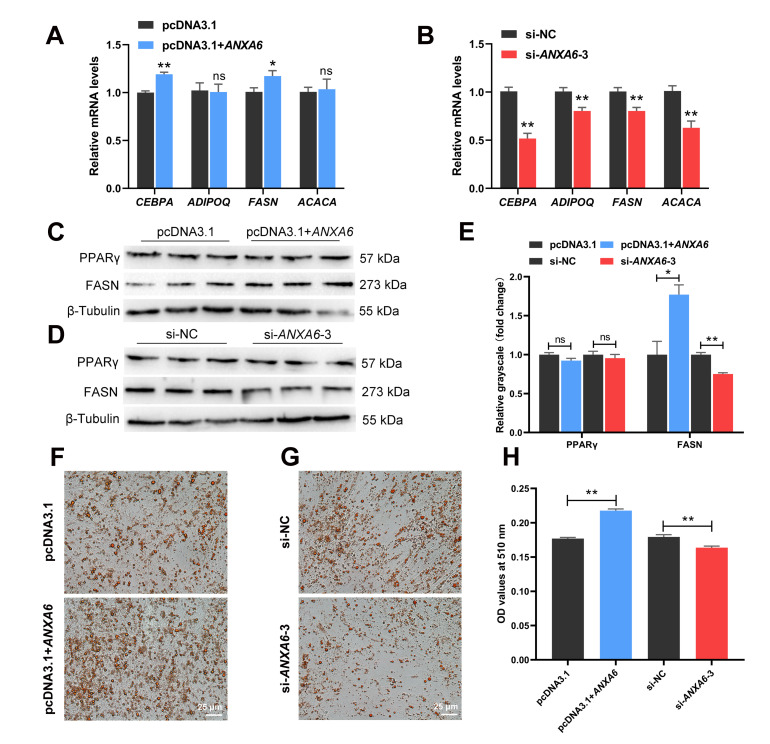
*ANXA6* promotes the differentiation of chicken intramuscular preadipocytes. (**A**,**B**) Relative mRNA levels of adipogenic genes (*CEBPA*, *ADIPOQ*, *FASN,* and *ACACA*) after overexpression or interference of *ANXA6*. (**C**,**D**) Relative protein levels of genes related to lipid metabolism (PPARγ and FASN) following overexpression or interference of *ANXA6*. (**E**) Relative grayscale of PPARγ and FASN proteins relative to β-Tubulin. (**F**,**G**) Lipid droplets stained with Oil red O in adipocytes following overexpression or interference of *ANXA6*. (**H**) Triglyceride contents measured by microplate reader after overexpression or interference of *ANXA6*. All results are presented as mean ± SEM. *n* = 3. * *p* < 0.05; ** *p* < 0.01; ^ns^ *p* > 0.05.

The expression of adipogenic genes (*CEBPA*, *ADIPOQ*, *FASN*, and *ACACA*) was measured in chicken intramuscular preadipocytes following transfection. The results demonstrated that the mRNA levels of *CEBPA* and *FASN* were ascended after *ANXA6* overexpression (Figure 3A), while *ANXA6* knockdown reduced the mRNA levels of *CEBPA*, *ADIPOQ*, *FASN*, and *ACACA* (Figure 3B). The protein levels of FASN also showed a similar trend (Figure 3C–E). However, PPARγ protein expression remained stable whether overexpression or interference of *ANXA6*. After preadipocytes were induced to differentiation for 10 d, we stained them with Oil red O, which showed that the formation of lipid droplets increased following *ANXA6* overexpression but decreased following *ANXA6* knockdown (Figure 3F,G). The determination of OD values at 510 nm further confirmed the above results (Figure 3H). All these results proved that *ANXA6* promotes the differentiation of chicken intramuscular preadipocytes.

### 3.4. ANXA6 Is a Target Gene of miR-24-3p

**Figure 4:**
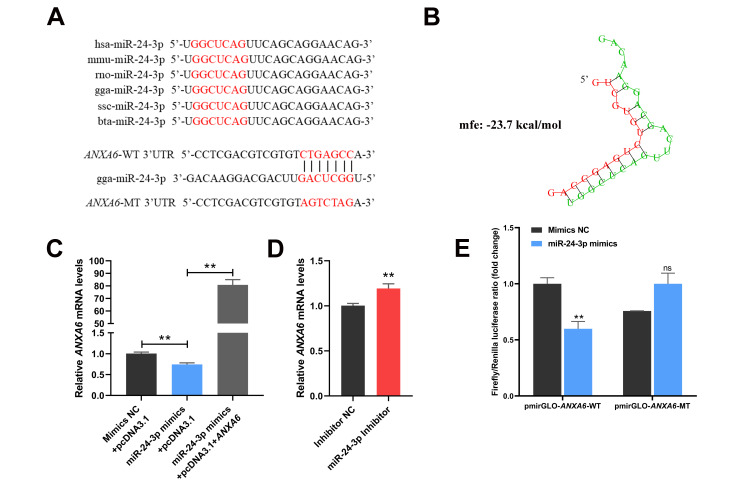
miR-24-3p regulates the expression of *ANXA6*. (**A**) Seed sequence of miR-24-3p in *Homo sapiens* (hsa), Mus musculus (mmu), *Rattus norvegicus* (rno), *Gallus gallus* (gga), *Sus scrofa* (ssc), and *Bos taurus* (bta). Wild type (WT) and mutant type (MT) of *ANXA6* binding site used for dual-luciferase reporter vector construction. (**B**) Complementary pattern diagram of miR-24-3p and *ANXA6* 3′UTR region. (**C**) Relative expression of *ANXA6* in preadipocytes after co-transfection of miR-24-3p mimics and *ANXA6* overexpression vector. (**D**) Relative expression of *ANXA6* after transfection with miR-24-3p inhibitor. (**E**) Relative luciferase activity of wild type (WT) or mutant type (MT) reporter vectors co-transfected with miR-24-3p mimic. All results are presented as mean ± SEM. *n* = 3. ** *p* < 0.01; ^ns^ *p* > 0.05.

Through applying miRDB software for bioinformatic analysis, we discovered that miR-24-3p is an upstream regulatory miRNA of *ANXA6*. The seed region of miR-24-3p is evolutionarily conserved and complementary to the 3′UTR of *ANXA6* (Figure 4A,B). The mRNA levels of *ANXA6* were decreased following transfection of miR-24-3p mimics but raised following transfection of both miR-24-3p mimics and *ANXA6* overexpression vector (Figure 4C) or miR-24-3p inhibitor (Figure 4D). Dual-luciferase reporter assay indicated that miR-24-3p mimics significantly restrained the luciferase activity of *ANXA6* wild-type plasmid compared with mimics NC (Figure 4E), presenting that miR-24-3p can directly bind to the 3′UTR of *ANXA6*.

### 3.5. miR-24-3p Promotes the Proliferation of Chicken Intramuscular Preadipocytes through Targeting ANXA6

**Figure 5:**
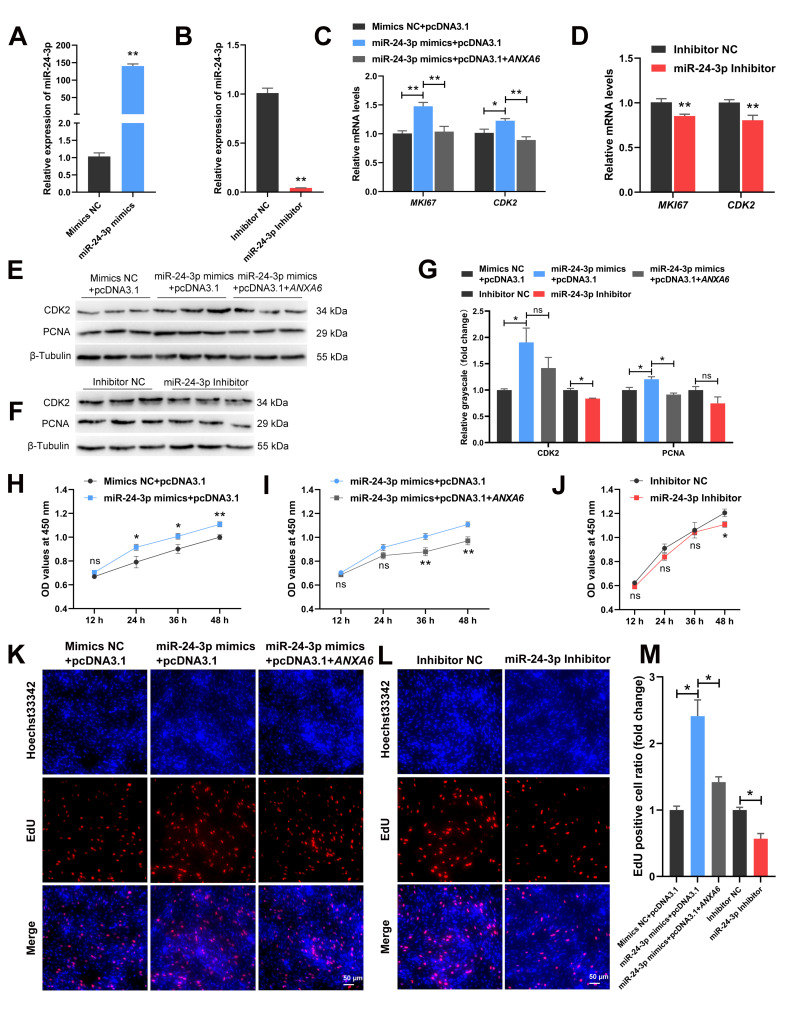
miR-24-3p promotes the proliferation of chicken intramuscular preadipocytes through targeting *ANXA6*. (**A**,**B**) Relative expression of miR-24-3p in preadipocytes transfected with mimics or inhibitor. (**C**,**D**) Relative mRNA levels of cell proliferation related genes (*MKI67* and *CDK2*) after co-transfection of miR-24-3p mimics and *ANXA6* overexpression vector or transfection with miR-24-3p inhibitor. (**E**,**F**) Relative expression of cell-cycle-associated protein (CDK2 and PCNA) following co-transfection of miR-24-3p mimics and *ANXA6* overexpression vector or transfection with miR-24-3p inhibitor. (**G**) Relative grayscale of CDK2 and PCNA proteins relative to β-Tubulin. (**H**–**J**) Cell growth curves determined by CCK-8 assay at 12 h, 24 h, 36 h, and 48 h following co-transfection of miR-24-3p mimics and *ANXA6* overexpression vector or transfection with the miR-24-3p inhibitor. (**K**,**L**) Proliferation state of preadipocytes assessed by EdU assay after co-transfection of miR-24-3p mimics and *ANXA6* overexpression vector or transfection with miR-24-3p inhibitor. (**M**) Relative EdU positive cell ratio following co-transfection of miR-24-3p mimics and *ANXA6* overexpression vector or transfection with the miR-24-3p inhibitor. All results are presented as mean ± SEM. *n* = 3. * *p* < 0.05; ** *p* < 0.01; ^ns^ *p* > 0.05.

To explore the effects of miR-24-3p on chicken intramuscular preadipocytes, the cells were transfected with miR-24-3p mimics or inhibitor. The expression of miR-24-3p was raised more than 140 times in the mimics group compared with the NC group (Figure 5A) and the interference efficiency achieved 95.7% (Figure 5B). The mRNA levels of *MKI67* and *CDK2* were increased following transfection of miR-24-3p mimics but decreased following transfection of both miR-24-3p mimics and *ANXA6* overexpression vector (Figure 5C) or miR-24-3p inhibitor (Figure 5D). The same phenomenon was observed in the protein levels of CDK2 and PCNA, although the change of CDK2 protein expression was not significant in the mimics group compared with co-transfection group and the change of PCNA protein expression was not significant in the inhibitor group compared with the NC group (Figure 5E–G). The CCK-8 assay showed that cell proliferation was promoted after transfection of miR-24-3p mimics (Figure 5H) but inhibited after transfection of both miR-24-3p mimics and *ANXA6* overexpression vector (Figure 5I) or miR-24-3p inhibitor (Figure 5J). EdU assay suggested that EdU positive cell ratio was raised following transfection of miR-24-3p mimics but reduced following transfection of both miR-24-3p mimics and *ANXA6* overexpression vector or miR-24-3p inhibitor (Figure 5K–M). Thus, we speculated that miR-24-3p promotes the proliferation of chicken intramuscular preadipocytes through targeting *ANXA6*.

### 3.6. miR-24-3p Inhibits the Differentiation of Chicken Intramuscular Preadipocytes through Targeting ANXA6

**Figure 6:**
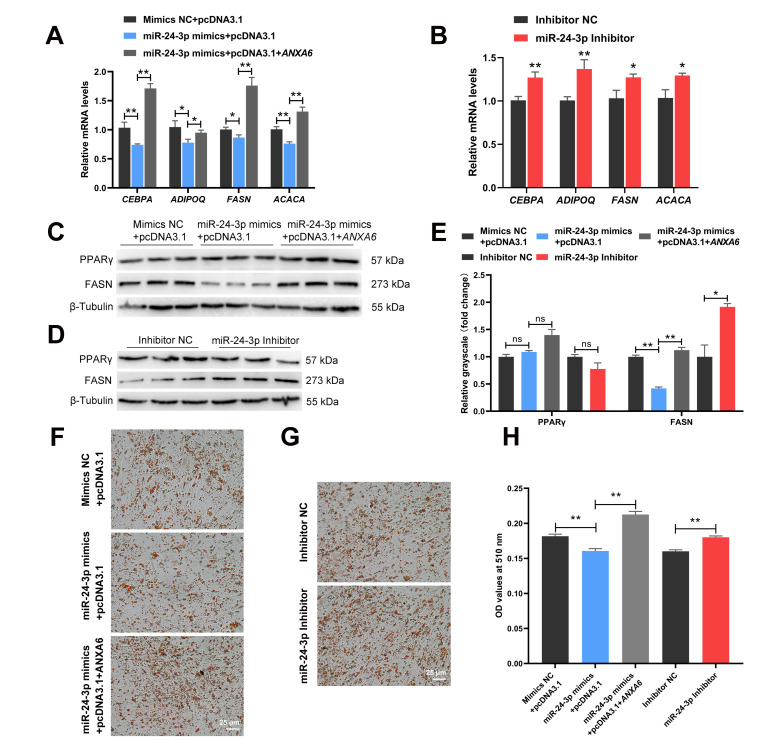
miR-24-3p inhibits the differentiation of chicken intramuscular preadipocytes through targeting *ANXA6*. (**A**,**B**) Relative mRNA levels of adipogenic genes (*CEBPA*, *ADIPOQ*, *FASN,* and *ACACA*) after co-transfection of the miR-24-3p mimics and the *ANXA6* overexpression vector or transfection with the miR-24-3p inhibitor. (**C**,**D**) Relative protein levels of genes related to lipid metabolism (PPARγ and FASN) following co-transfection of the miR-24-3p mimics and the *ANXA6* overexpression vector or transfection with the miR-24-3p inhibitor. (**E**) Relative grayscale of PPARγ and FASN proteins relative to β-Tubulin. (**F**,**G**) Lipid droplets stained with Oil red O in adipocytes following co-transfection of the miR-24-3p mimics and the *ANXA6* overexpression vector or transfection with the miR-24-3p inhibitor. (**H**) Triglyceride contents measured by microplate reader after co-transfection of the miR-24-3p mimics and the *ANXA6* overexpression vector or transfection with the miR-24-3p inhibitor. All results are presented as mean ± SEM. *n* = 3. * *p* < 0.05; ** *p* < 0.01; ^ns^ *p* > 0.05.

The qPCR results demonstrated that the expression of *CEBPA*, *ADIPOQ*, *FASN,* and *ACACA* were inhibited following transfection of the miR-24-3p mimics but promoted following transfection of both the miR-24-3p mimics and *ANXA6* overexpression vector (Figure 6A) or the miR-24-3p inhibitor (Figure 6B). The protein levels of FASN showed the same trend as the above results (Figure 6C–E). Nevertheless, the protein levels of PPARγ were not significantly influenced whether transfection of the miR-24-3p mimics, both the miR-24-3p mimics and the *ANXA6* overexpression vector, or the miR-24-3p inhibitor. Oil red O staining presented that the formation of lipid droplets decreased after transfection of the miR-24-3p mimics but decreased after transfection of both the miR-24-3p mimics and the *ANXA6* overexpression vector (Figure 6F) or the miR-24-3p inhibitor (Figure 6G), which was in accordance with the results of the OD value determination at 510 nm (Figure 6H).

## 4. Discussion

IMF content of farm animals is influenced by heredity, nutrition, and management [16]. Among genetic factors, genes can precisely regulate IMF accumulation in skeletal muscle. For instance, *AQP3* promoted the proliferation and differentiation of porcine intramuscular preadipocytes [17]. The lipid synthesis was inhibited both in porcine subcutaneous and intramuscular preadipocytes after knockdown of *CTRP6* [18]. Overexpression of *KLF9* inhibited the expression of the adipogenic marker gene *AP2* in chicken intramuscular preadipocytes, while knockout of *KLF9* promoted the expression of *PPARγ*, *CEBPA,* and *AP2* as well as the accumulation of triglycerides [19]. Downregulation of *SLC27A1* expression inhibited *CPT1A*-mediated fatty acid oxidation, thereby increasing IMF deposition in chickens [20]. Our results indicated that *ANXA6* was highly expressed in adipose tissue compared with others, and its expression rose significantly on the second day of intramuscular preadipocyte differentiation, suggesting that *ANXA6* may be a key regulatory gene of IMF deposition.

Research on the function of *ANXA6* mainly concentrate on many cancers. *ANXA6* promoted autophagy by inhibiting the PI3K/AKT/mTOR signaling pathway in nasopharyngeal carcinoma [21]. *ANXA6* suppressed the tumorigenesis of cervical cancer via inducting autophagy [22]. However, the role of *ANXA6* in livestock production remains unclear. In this study, we revealed that *ANXA6* inhibited proliferation and promoted differentiation of chicken intramuscular preadipocytes to dominate IMF deposition. Previous studies showed that *ANXA6*-knockout mice gained less weight compared with controls during the course of high-fat diet feeding [23], which proved *ANXA6* conduced to fat deposition and coincided with our results. *PPARγ*, a classic promoter gene of lipid metabolism [24], was not influenced after *ANXA6* overexpression or interference. Therefore, *ANXA6* regulated the formation of IMF by acting on *CEBPA*, *ADIPOQ*, *FASN*, and *ACACA* rather than *PPARγ* on the basis of our results.

miR-24-3p functions through blocking target gene expression in numerous biological processes including cell migration, proliferation, apoptosis, and differentiation. For the reason that miR-24-3p is evolutionarily conserved, its regulatory effects are theoretically similar in different species. In recent studies, miR-24-3p was proved to promote proliferation and inhibit apoptosis of C2C12 cells by targeting *CAMK2B* [25]. Moreover, miR-24-3p promoted cell migration and proliferation in lung cancer by targeting *SOX7* [26]. Our results demonstrated that miR-24-3p promoted proliferation of chicken intramuscular preadipocytes. Nevertheless, miR-24-3p was also reported to inhibit the proliferation of fetal bovine skeletal muscle-derived progenitor cells through targeting *ACVR1B* [27]. This phenomenon may be caused by different cell types. In addition, we revealed that miR-24-3p contributed to the differentiation of chicken intramuscular preadipocytes and subsequent lipid deposition, which was consistent with other studies [11,12].

Undoubtedly, a growing number of miRNAs were confirmed to participate in the IMF accumulation process. While miR-29b/c [28] and miR-125a-5p [29] promoted proliferation and inhibited differentiation of porcine intramuscular preadipocytes, miR-146a-5p [30], and miR-425-5p [31] inhibited both proliferation and differentiation. The differentiation of chicken intramuscular preadipocytes was boosted by miR-15a [32] but restrained by miR-18b-3p [33] and miR-223 [34]. In the current study, we determined that miR-24-3p transformed the regulatory effects of *ANXA6* on intramuscular preadipocytes by blocking its expression.

## 5. Conclusions

**Figure 7:**
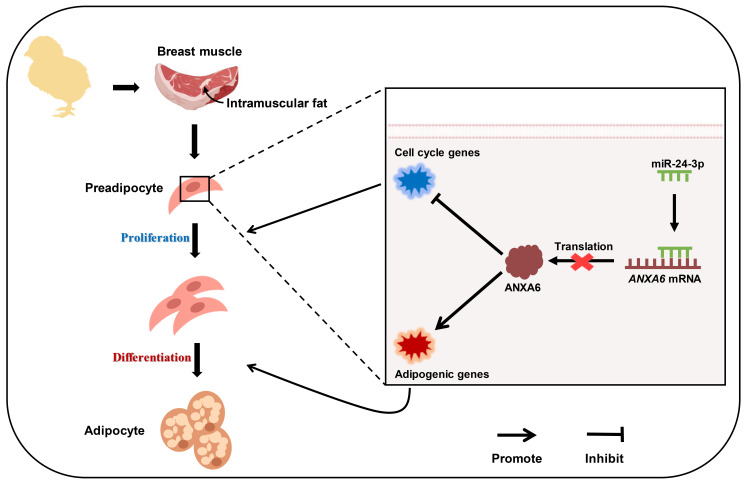
A schematic model depicting the role of miR-24-3p and *ANXA6* in regulating chicken intramuscular fat deposition. miR-24-3p promotes proliferation and inhibits differentiation of chicken intramuscular preadipocytes by blocking *ANXA6* expression.

In conclusion, our results revealed that *ANXA6* inhibited proliferation and promoted differentiation of chicken intramuscular preadipocytes, but miR-24-3p reversed the effects of *ANXA6* on preadipocyte proliferation and differentiation by combining to 3′UTR of *ANXA6*, and then influenced the accumulation of intramuscular fat (Figure 7). These findings may contribute to furnish new insights for the improvement of meat quality in broilers.

## Data Availability

Not applicable.

## Supplementary Materials

The following supporting information can be downloaded at: https://www.mdpi.com/article/10.3390/genes13040635/s1, Table S1: The RNA oligonucleotides used for cell transfection; Table S2: The specific primers used for qPCR.
