# Supplementary material for: miR-24-3p Dominates the Proliferation and Differentiation of Chicken Intramuscular Preadipocytes by Blocking ANXA6 Expression
Source: Genes (Basel). 2022 Apr 2;13(4):635. doi: 10.3390/genes13040635 (PMC9024460; doi:10.3390/genes13040635)
Supplement: Supplementary file 1 [file genes-13-00635-s001.zip › genes-1661295-supplementary.pdf]

Table S1. The RNA oligonucleotides used for cell transfection.

Name	Sequence (5’→3’)
si-NC	F: UUCUCCGAACGUGUCACGUTT
	R: ACGUGACACGUUCGGAGAATT
si-*ANXA6*-1	F: GGCUGAUUGUAAGCUUGAUTT
	R: AUCAAGCUUACAAUCAGCCTT
si-*ANXA6*-2	F: CUCUCCACAAUAUGAUAAATT
	R: UUUAUCAUAUUGUGGAGAGTT
si-*ANXA6*-3	F: GGCCUACAAAGCUCACUAUTT
	R: AUAGUGAGCUUUGUAGGCCTT
Mimics NC	F: UUCUCCGAACGUGUCACGUTT
	R: ACGUGACACGUUCGGAGAATT
miR-24-3p mimics	F: UGGCUCAGUUCAGCAGGAACAG
	R: GUUCCUGCUGAACUGAGCCAUU
Inhibitor NC	CAGUACUUUUGUGUAGUACAA
miR-24-3p inhibitor	CUGUUCCUGCUGAACUGAGCCA

Table S2. The specific primers used for qPCR.

Accession No.	Gene symbol	Primer sequence (5'→3')	Product length (bp)
NM_001397313.1	*ANXA6*	F: ATGGCACCCAAAGGAAAGGT<br>R: GTACAAGGCGTCTGCATCCT	84
XM_015289038.2	*MKI67*	F: GCAACAACAAGGAGGCTTCG<br>R: TTCAGGTGCCATCCCGTAAC	204
NM_001199857.1	*CDK2*	F: GCTCTTCCGTATCTTCCGCA<br>R: ATGCGCTTGTTGGGATCGTA	192
NM_001031459.1	*CEBPA*	F: GGAGCAAGCCAACTTCTACG<br>R: GAGTGCTCGTTCTCGCAGAT	174
NM_206991.1	*ADIPOQ*	F: GCAGAACCACTACGACAGCA<br>R: TAGACCCCGTTGTTGTTGCC	258
NM_205155.3	*FASN*	F: ACACCCTAAGCCTCGTTC<br>R: CCTCAAGATAGCCTGTAAGA	208
NM_205505.1	*ACACA*	F: TCCTGCCTGCTCATACTT<br>R: GCGATACCTGTCCACTTC	227
NM_204305.1	*GAPDH*	F: GGGGAAAGTCATCCCTGAGC<br>R: AGCAGCCTTCACTACCCTCT	145
-	miR-24-3p	F: TGGCTCAGTTCAGCAGGAACAG<br>R: CAGGTCCAGTTTTTTTTTTTTTT	-
-	U6	F: GGGCCATGCTAATCTTCTCTGTA<br>R: CAGGTCCAGTTTTTTTTTTTTTT	-
